# Supplementary material for: NSD2 overexpression drives clustered chromatin and transcriptional changes in a subset of insulated domains
Source: Nat Commun. 2019 Oct 24;10:4843. doi: 10.1038/s41467-019-12811-4 (PMC6813313; doi:10.1038/s41467-019-12811-4)
Supplement: Supplementary file 2 — Supplementary Information [file 41467_2019_12811_MOESM2_ESM.pdf]

NSD2 overexpression drives clustered chromatin and transcriptional changes in a subset of insulated domains. Lhoumaud et al.

Supplementary Figures 1-9

Supplementary Tables 1-2

Supplementary References for Methods

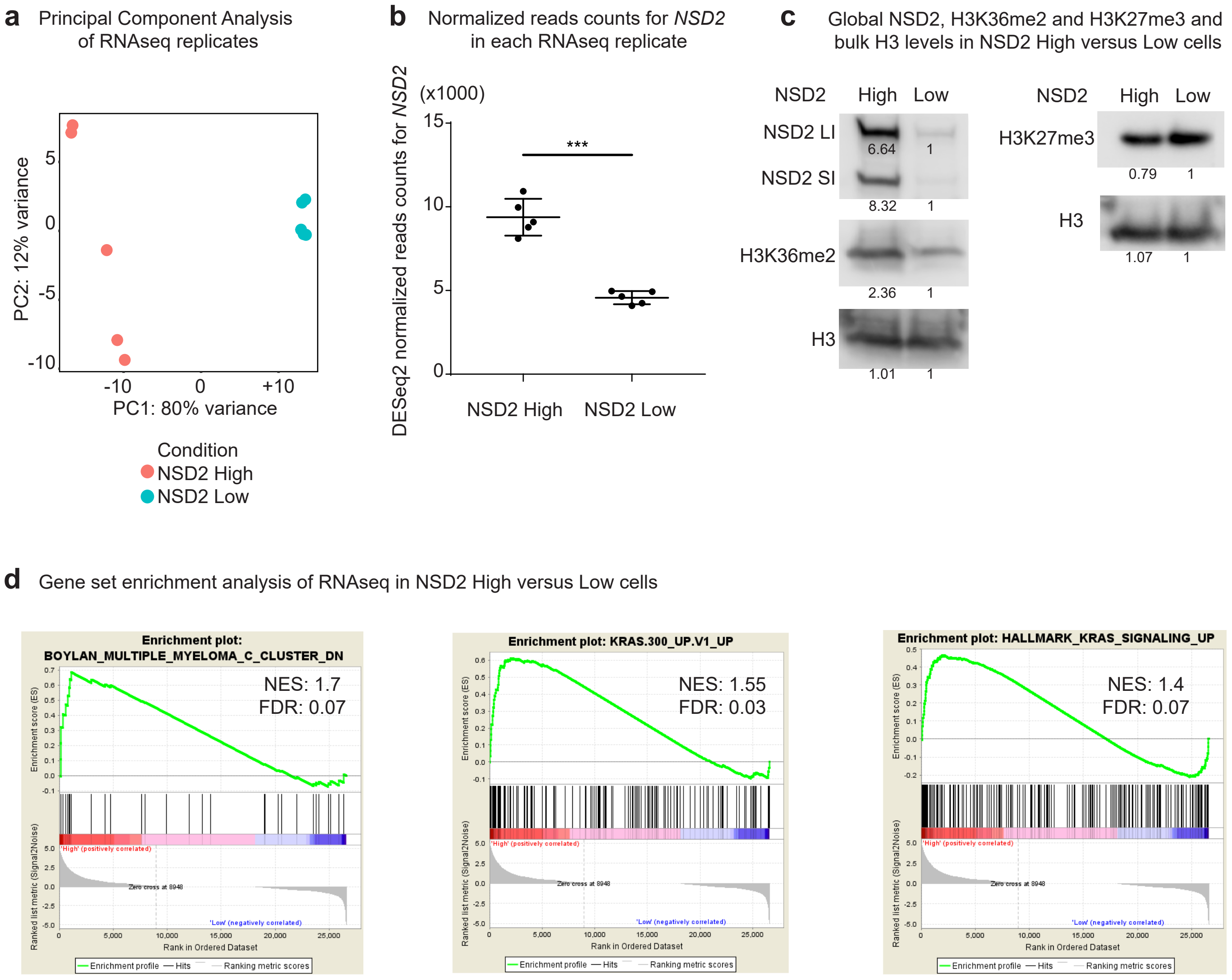

Supplementary Figure 1. RNAseq analysis in NSD2 High versus Low cells. a, PCA of RNA-seq replicates for NSD2 High and Low cells, n=5 independent experiments. **b,** Normalized read counts for NSD2 in each RNAseq replicate (n=5 independent experiments, *** p <0.0001, t-test). Each dot represents as replicate and mean with SD (standard deviation) are shown. **c,** Western blot showing global levels of NSD2, H3K36me2 and H3K27me3 in NSD2 High versus Low cells as compared to bulk H3, which is used as loading control. Numbers below the signal indicate the quantification performed using ImageJ and normalized by the NSD2 low condition. LI: long isoform (155 kDa). SI: short isoform (75 kDa). Uncropped plots with ladder marks are provided in Source Data file. **d,** Enrichment of MM and KRAS pathways as shown by GSEA based on the fold change of expression in NSD2 high vs low cells across all genes (13603 genes, see Method for details). NES: normalized enrichment score.

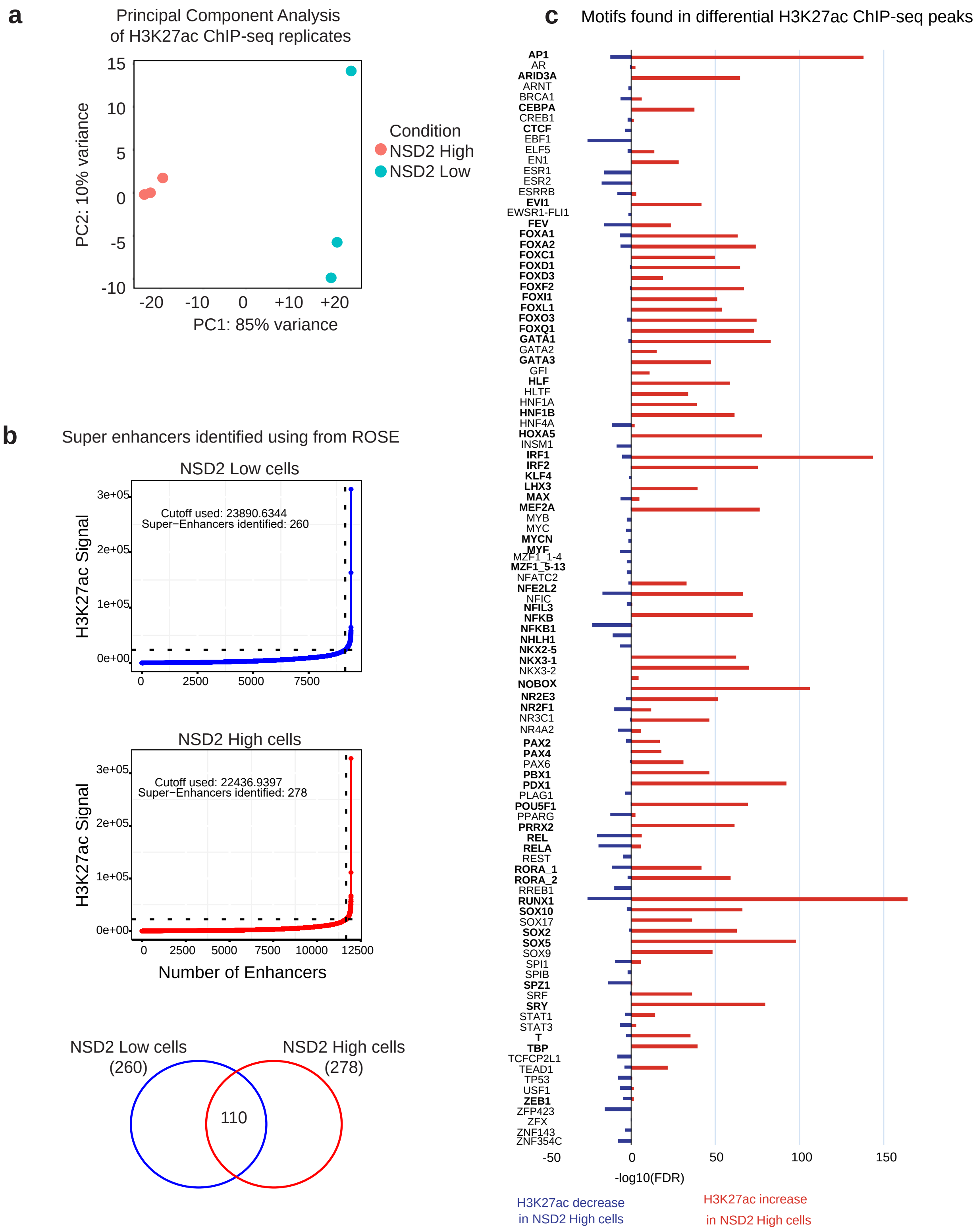

Supplementary Figure 2. Transcription factor motifs identified in differential H3K27ac peaks. a, PCA of H3K27ac ChIP-seq replicates for NSD2 High and Low cells, n=3 independent experiments. b, Identification of super-enhancers in NSD2 Low (upper panel) and High (middle panel) using H3K27ac with 'ROSE' (rank ordering of super-enhancers[1]). The overlap between NSD2 Low versus High is shown in the lower panel. c, Transcription factor motifs identified in increased (1650) and decreased (303) H3K27ac peaks using TRAP, related to Fig. 1. Motifs also presented in Fig. 1f are in bold. Motifs found in increased (red) and decreased (blue) H3K27ac peaks (-log10 FDR).

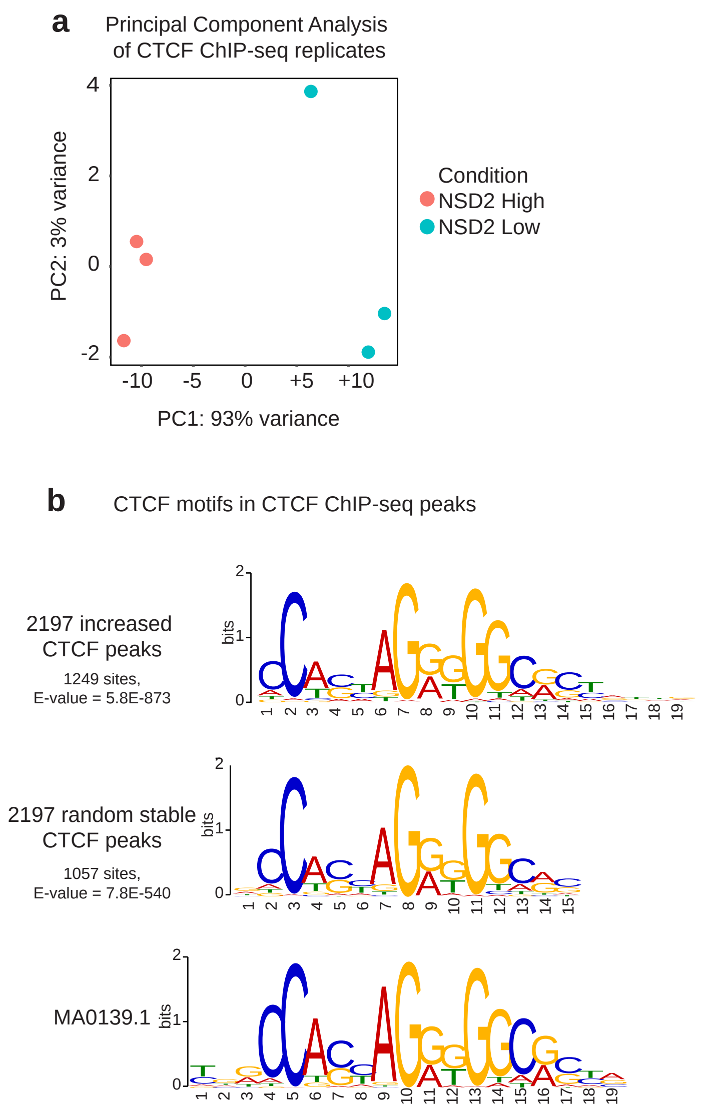

Supplementary Figure 3. CTCF peaks changes and associated motifs. a, PCA of CTCF ChIP-seq replicates for NSD2 High and Low cells, n=5 independent experiments. **b,** CTCF motifs in CTCF ChIP-seq peaks identified using MEME-ChIP tool from the MEME suite[2]. 2197 increased peaks from **Fig. 2a**, (top panel), 2197 randomly selected CTCF peaks from the full list of peaks, (middle panel) and the CTCF motif MA0139.1 from the Jaspar database (bottom panel). The numbers of CTCF sites and the E-value from MEME-ChIP are indicated.

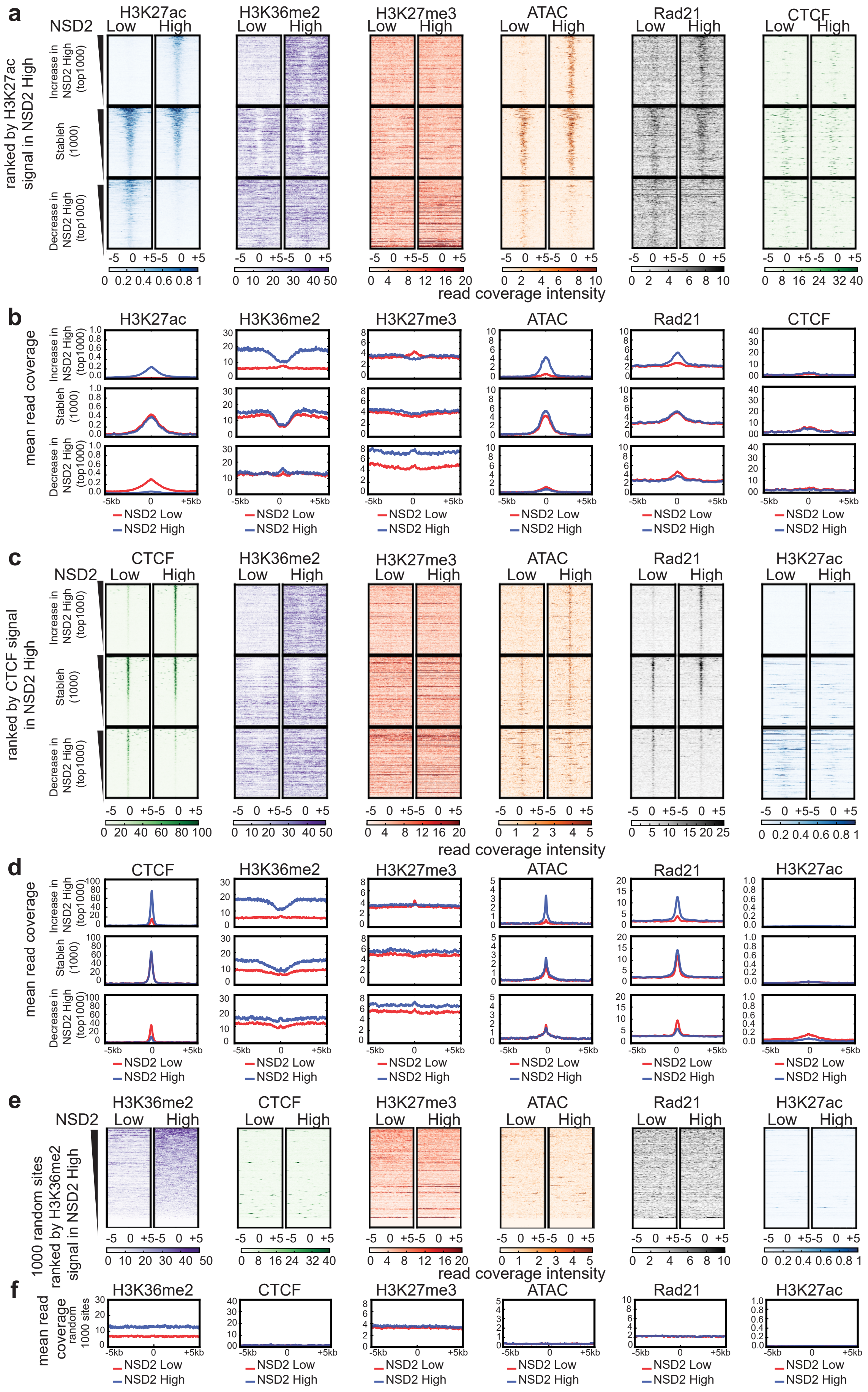
a
NSD2
H3K27ac
H3K36me2
H3K27me3
ATAC
Rad21
CTCF
Low
High
ranked by H3K27ac signal in NSD2 High
Increase in NSD2 High (top1000)
Stableh (1000)
Decrease in NSD2 High (top1000)
read coverage intensity
b
mean read coverage
NSD2 Low
NSD2 High
-5kb
0
+5kb
c
ranked by CTCF signal in NSD2 High
d
e
1000 random sites ranked by H3K36me2 signal in NSD2 High
f
mean read coverage random 1000 sites

Supplementary Figure 4. Chromatin landscape of the regions surrounding CTCF and H3K27ac changes. Heatmaps **a,** and average profiles **b,** of H3K27ac, H3K36me2, H3K27me3, ATAC-seq, Rad21 and CTCF signal at the top 1000 increased, 1000 stable and 1000 decreased H3K27ac peaks in NSD2 High versus Low cells. Heatmaps **c,** and average profiles **d,** of CTCF, H3K36me2, H3K27me3, ATAC-seq, Rad21 and H3K27ac signal at top 1000 increased, 1000 stable and 1000 decreased CTCF peaks in NSD2 High versus Low cells. Top 1000 increased, 1000 stable and 1000 decreased CTCF and H3K27ac peaks are based on Log2 Fold changes in reads counts in NSD2 High versus Low cells. Peaks are ranked by H3K27ac **a,** and CTCF **c,** signal in NSD2 High cells. Heatmaps **e,** and average profiles **f,** of CTCF, H3K36me2, H3K27me3, ATAC-seq, Rad21 and H3K27ac signal at 1000 randomly picked sites.

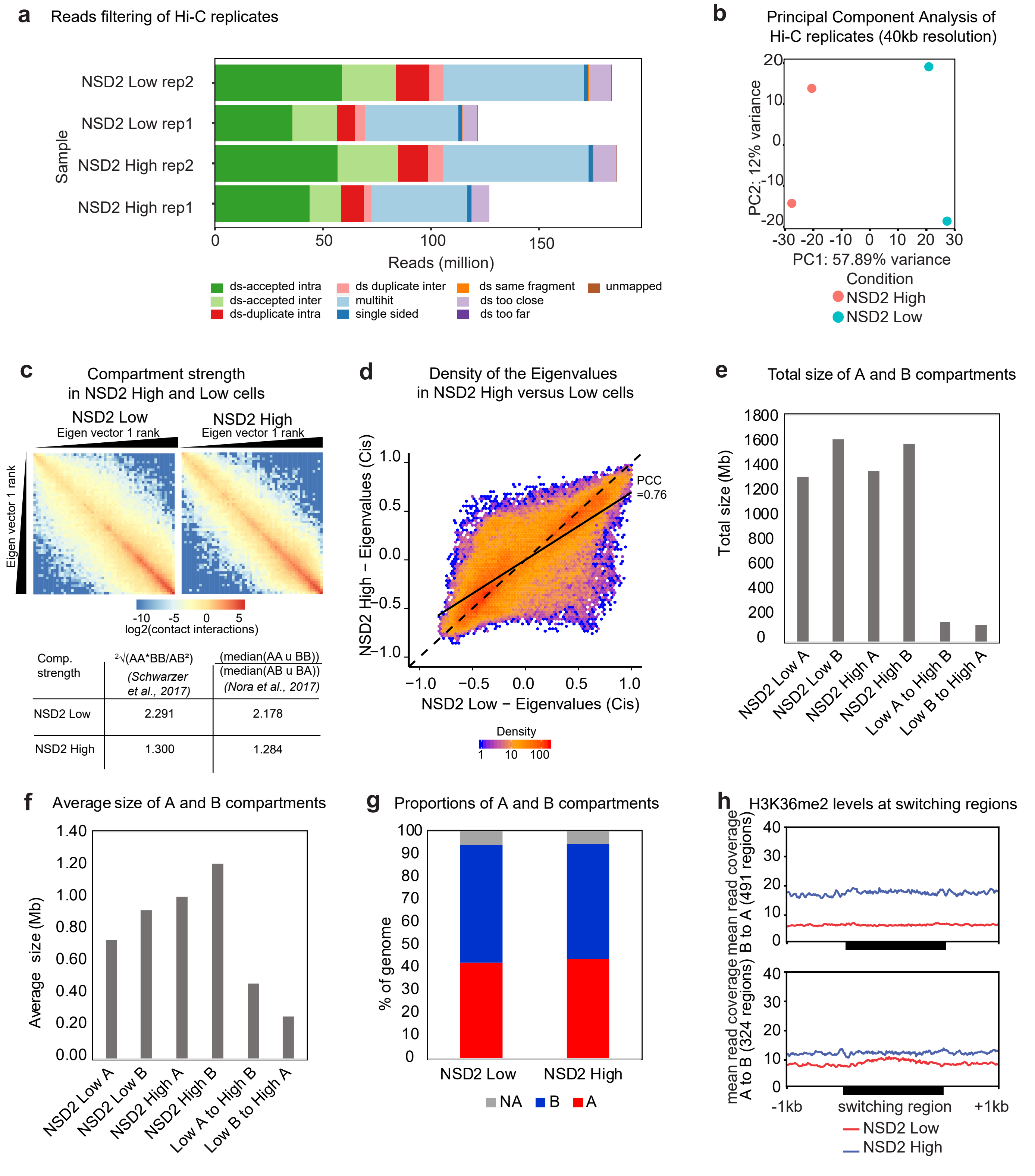

Supplementary Figure 5. Compartment analysis in NSD2 High and Low cells using Hi-C. a, Histogram representing read filtering of Hi-C duplicates processed using Hi-C bench[3]. 'Ds accepted-intra' and 'ds accepted inter' reads were retained for further analysis. **b,** PCA of Hi-C replicates for NSD2 High and Low cells processed with Hi-C bench at a resolution of 40 kb, n=2 independent experiments. **c,** Compartmentalization saddle plots of the cis-eigenvector 1 values (50kb bins) in 50 equally sized ranks (pooled replicates) sorted in ascending compartment signal values (strongest B-compartment like bins to strongly A-compartment like bins) and computed

pairwise log2 enrichment of contact counts (IC-normalized) between all groups (from left to right and from top to bottom: the 50 groups of eigenvalue bins ranked from strongest B-like to strongest A-like bins) (top panel) and compartment strength values (bottom panel) calculated as per Nora et al.,[4] and Schwarzer et al.,[5]. **d,** Density of the Eigenvalues in NSD2 High versus Low cells. PCC refers to Pearson correlation curve as compared to the dotted line reference. Source data are provided as a Source Data file. Histograms representing the total size **e,** and average size **f,** of A, B and switching compartments in NSD2 High and Low cells. **g,** Bar plot representing the proportion of A (red) and B (blue) compartments in the genome for NSD2 High and Low cells. **h,** H3K36me2 average profiles at regions switching from B to A (491 regions, top panel) and from A to B (324 regions, bottom panel).

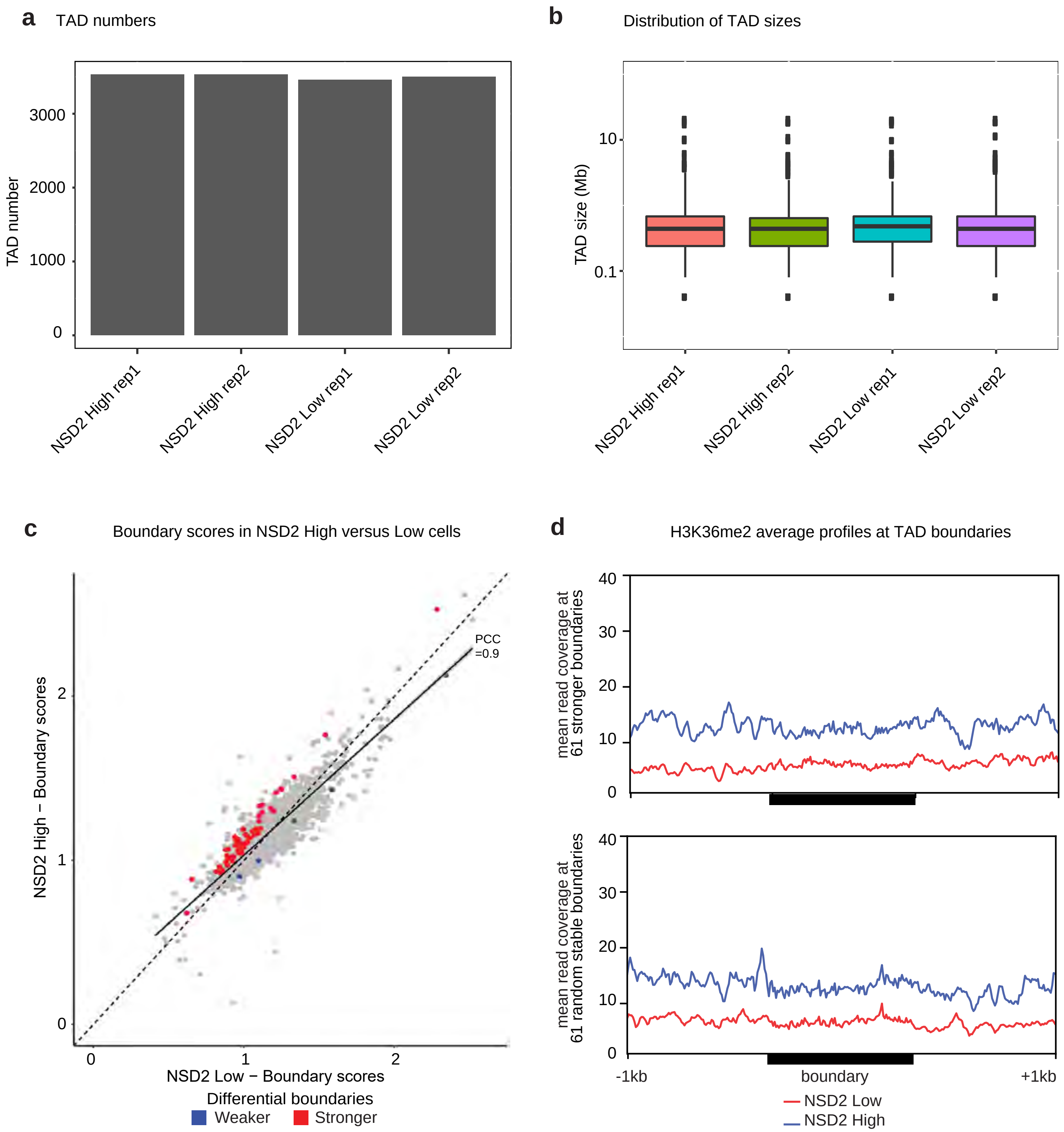

Supplementary Figure 6. TAD analysis in NSD2 High and Low cells using Hi-C. a, Histogram representing the number of TADs for each replicate of NSD2 High and Low cells. **b,** Boxplots representing the distribution of TAD sizes (in megabases, Mb), for each replicate of NSD2 High and Low cells. Center lines represent the medians. **c,** Boundary scores in NSD2 High versus Low cells. NSD2 overexpression is associated with increases (red, 61) and decreases (blue, 5) in TAD boundary strength (cutoffs of absolute Log2 fold change > 0.1 and FDR < 0.05, see **Fig. 4a**). PCC refers to Pearson correlation curve as compared to the dotted line reference. **d,** H3K36me2 average profiles at TAD boundaries, for the 61 boundaries that gain strength in NSD2 High cells (top panel) and 61 random stable boundaries (bottom panel). Source data are provided as a Source Data file.

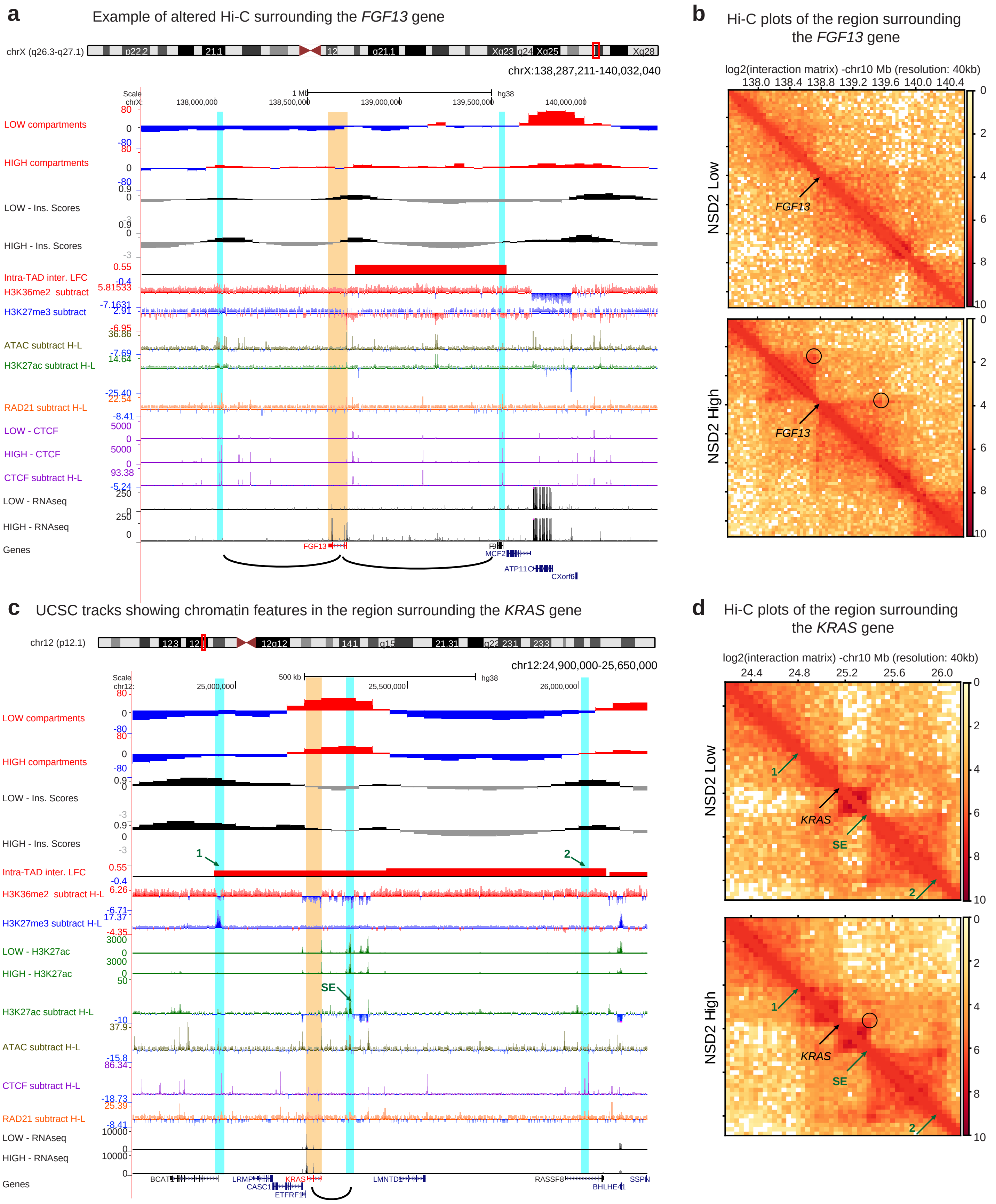

Supplementary Figure 7. Examples of altered chromatin at oncogenes. a, UCSC tracks showing chromatin features in the region surrounding the *FGF13* gene (*FGF13* gene indicated in red and location highlighted by a yellow stripe) and the new contacts that are formed by stronger CTCF and Rad21 binding (blue stripes and shown as a loop below). H-L refers to subtraction of the ChIP-seq signal (NSD2 High - Low).

b, Hi-C plots of the region surrounding the *FGF13* gene. Top panel: NSD2 Low, bottom panel: NSD2 High. Black arrow indicates the *FGF13* gene. Circle indicates a loop between *FGF13* and surrounding increased CTCF and Rad21 regions highlighted by a blue strip in panel **b**. **c,** UCSC tracks showing chromatin features in the region surrounding the *KRAS* gene (*KRAS* gene indicated in red and highlighted by a yellow stripe) and a downstream super enhancer (indicated by a green arrow and highlighted by a blue stripe). Left and right boundaries surrounding intra-TAD interaction gain are highlighted by blue stripes and indicated by green arrows labelled as boundary '1' and '2', respectively. A graphical representation of the interaction between *KRAS* and the super enhancer is shown as a loop below. H-L refers to subtraction of the ChIP-seq signal (NSD2 High - Low). **d,** Hi-C plots of the region surrounding the *KRAS* gene in NSD2 Low versus High cells (top and bottom panels, respectively). Black arrow indicates the location of *KRAS*. Green arrows indicate the super enhancer (also labelled as 'SE'), and left and right boundaries surrounding intra-TAD interaction gain as labelled as boundary '1' and '2', respectively. Circle indicates a loop between *KRAS* and the super enhancer.

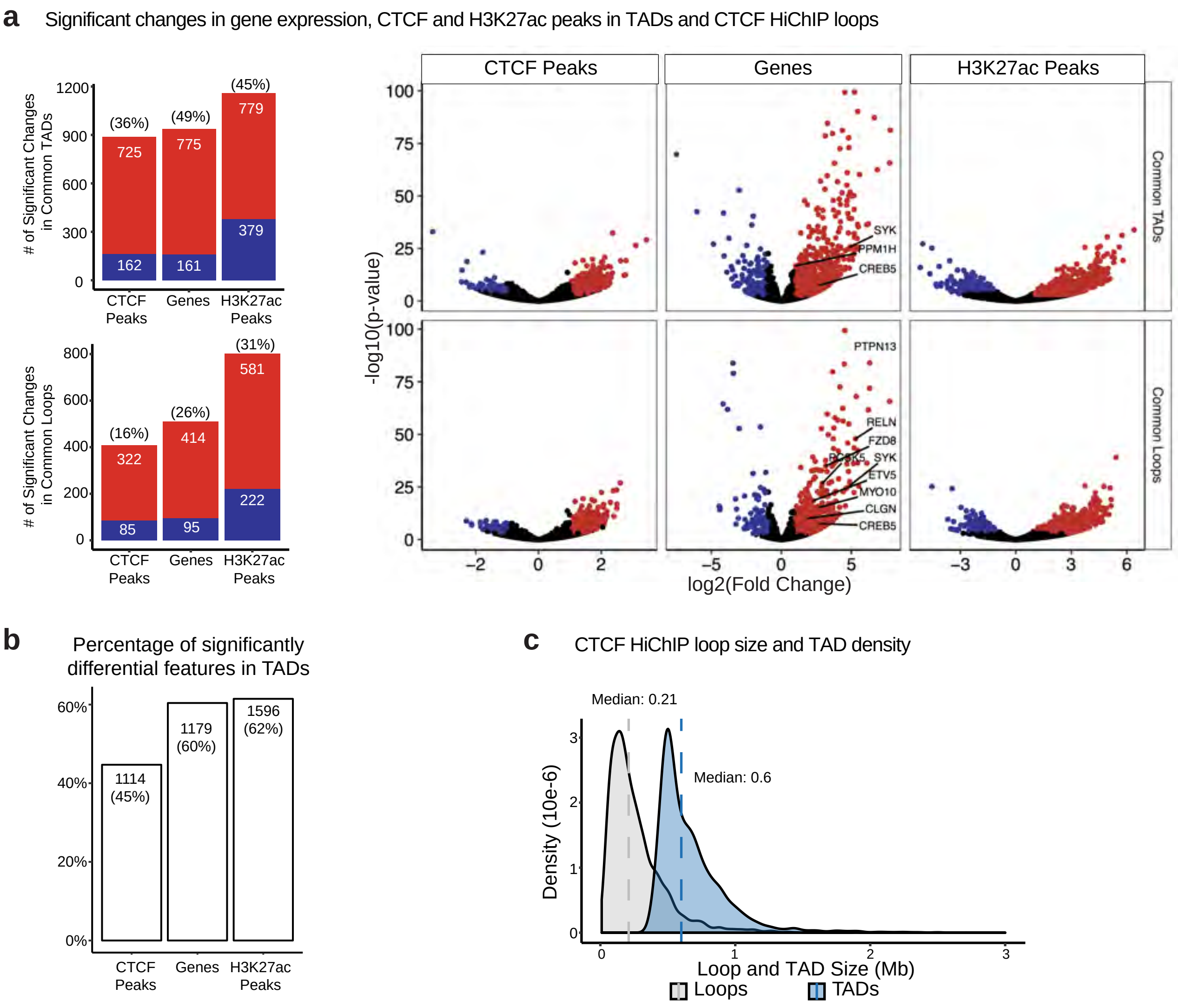

Supplementary Figure 8. NSD2 overexpression drives concordant chromatin and transcriptional changes in insulated domains. a, Bar plot (left panel) and Volcano plots (right panel) showing significant NSD2-mediated changes in CTCF binding (left panels), gene expression (middle panels) and H3K27ac signal (right panels) within TADs (top panels) or CTCF HiChIP loops (bottom panels). Increases of log2 fold change >1 are shown in red and decreases, log2 fold change <-1 are shown in blue (FDR <0.01). **b,** Percentage of significantly differential features in TADs. **c,** Density plot of CTCF HiChIP loop sizes.

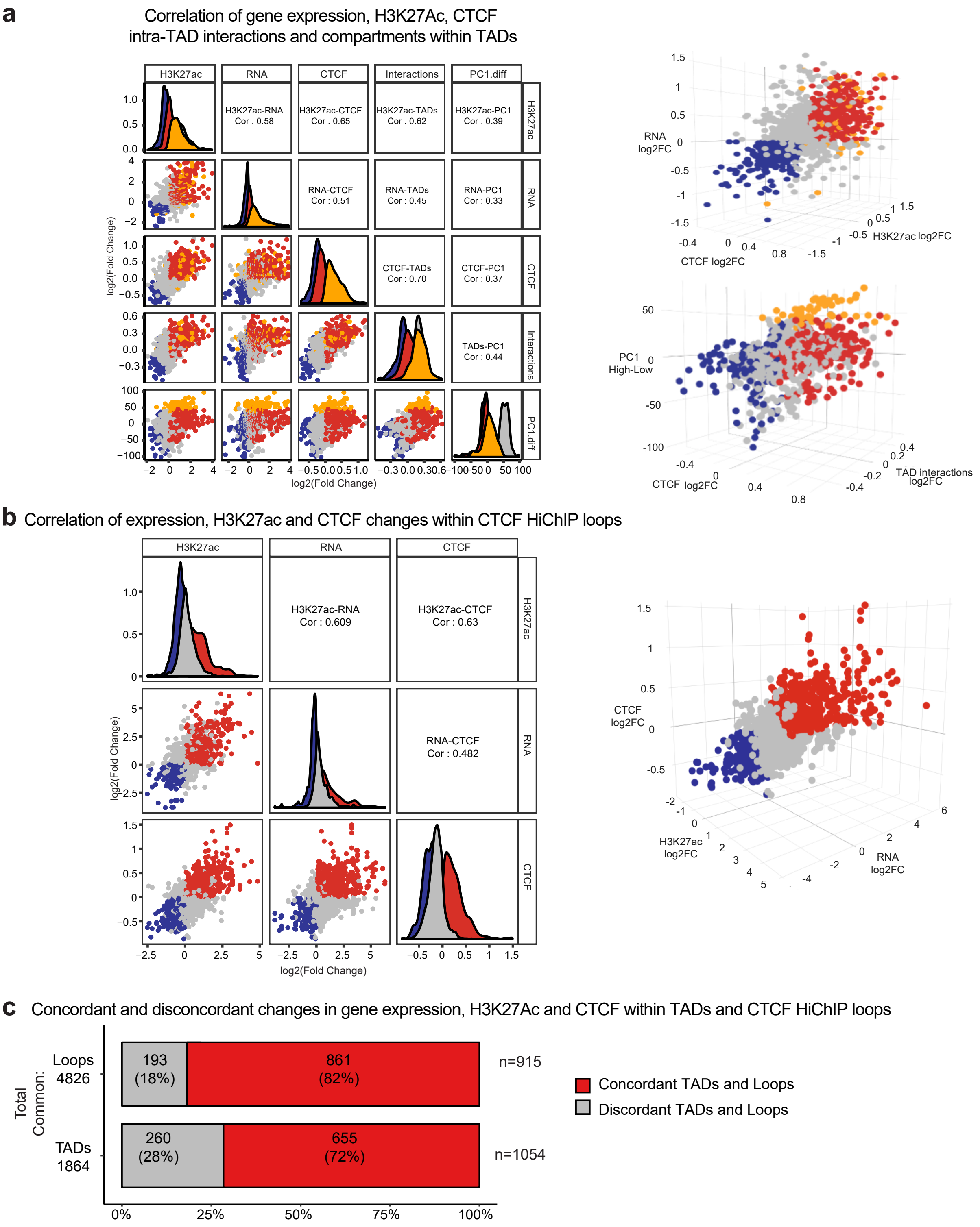

Supplementary Figure 9. Concordant chromatin and transcriptional changes in insulated domains. Pairwise (2D scatter plots left panel) and three-way (3D scatter plots right panels) comparisons representing log2 fold-changes of gene expression, H3K27ac, CTCF, intra-TAD interactions and PC1 values (representing subtraction of NSD2 High and Low levels) within **a,** TADs and **b,** CTCF HiChIP loops in NSD2 High versus Low cells. Concordant increased and decreased changing TADs are colored in red and blue, respectively. TADs that switch from B to A as determined by HOMER analysis (see method for details) are highlighted in orange. Pearson correlations are indicated. **c,** Bar graph showing the proportion of concordant and discordant changes in CTCF, H3K27ac and gene expression in TADs and CTCF loops. Source data for panels **a,** and **b,** are provided as a Source Data file.

sample	cell line	condition	experiments
KMS11	KMS11	NSD2 High	RNAseq, H3K27ac ChIPmentation, CTCF ChIPmentation, H3K36me2 ChIPmentation, CTCF HiChIP
NTKO rep1	NTKO	NSD2 High	RNAseq, H3K27ac ChIPmentation, CTCF ChIPmentation, H3K36me2 ChIPmentation, Rad21 ChIPmentation, Hi-C, 4C-seq, CTCF HiChIP
NTKO rep2	NTKO	NSD2 High	RNAseq, H3K27ac ChIPmentation, CTCF ChIPmentation, Rad21 ChIPmentation, Hi-C, 4C-seq, CTCF HiChIP
NTKO rep3	NTKO	NSD2 High	RNAseq, ATAC-seq
NTKO rep4	NTKO	NSD2 High	RNAseq, ATAC-seq
TKO clone 1 rep1	TKO	NSD2 Low	RNAseq, H3K27ac ChIPmentation, CTCF ChIPmentation, H3K36me2 ChIPmentation, Rad21 ChIPmentation, Hi-C, 4C-seq, CTCF HiChIP
TKO clone 1 rep2	TKO	NSD2 Low	RNAseq, ATAC-seq
TKO clone 2 rep1	TKO	NSD2 Low	RNAseq, H3K27ac ChIPmentation, CTCF ChIPmentation, H3K36me2 ChIPmentation, Rad21 ChIPmentation, Hi-C, 4C-seq, CTCF HiChIP
TKO clone 2 rep2	TKO	NSD2 Low	RNAseq, H3K27ac ChIPmentation, CTCF ChIPmentation, CTCF HiChIP
TKO clone 2 rep3	TKO	NSD2 Low	RNAseq, ATAC-seq

Supplementary Table 1: samples, cell lines and condition for each experiment

Analysis	Figure	Test/tool	Default	Cutoff	Remarks
Differentially expressed genes	1b	DEseq2 Wald test	NA	FDR < 0.01	
NSD2 normalized reads counts	Supp 1b	t-test	NA	***p-value<0.0001	
Gene set enrichment analysis of RNAseq in NSD2 High versus Low cells	Supp 1d	GSEA	yes	FDR < 0.25	
Differential H3K27ac peaks	1c	DEseq2 Wald test	NA	FDR< 0.01	
Differential H3K27ac at super-enhancers	1e	DEseq2 Wald test	NA	FDR < 0.1	a less stringent cutoff was used as compared to differential H3K27ac as overall changes are more modests at super-enhancers. Applying a cutoff of FDR < 0.01 would lead to only 30 significant SE
Motifs found in ATAC-seq peaks	1f and Supp. 2c	TRAP	yes	FDR < 0.01	
Promoter and distal H3K27ac and SE are associated with gene expression	1g	wilcoxon	NA	*p-value<0.05, **p-value<0.01, ***p-value<0.001	
Differential CTCF peaks	2a	DEseq2 Wald test	NA	FDR < 0.01	
Promoter and distal CTCF are associated with gene expression	2c	wilcoxon	NA	*p-value<0.05, **p-value<0.01, ***p-value<0.001	
Chromatin features at switching compartments	3e	wilcoxon	NA	*p-value<0.05, **p-value<0.01, ***p-value<0.001	
Boundary alterations in NSD2 High versus Low cells	4a	HiC bench	Yes	FDR < 0.05	
Boundary alterations correlate with CTC and Rad21 binding	4b	wilcoxon	NA	*p-value<0.05, **p-value<0.01, ***p-value<0.001	
Differential intra-TAD interactions in NSD2 High versus Low cells	4c	HiC bench	Yes	FDR < 0.05	
H3K36me2 and H3K27me3 in TADs	4d	wilcoxon	NA	*p-value<0.05, **p-value<0.01, ***p-value<0.001	
4C at SYK locus	6b	DEseq2 Wald test	NA	FDR < 0.01	
Differentially expressed genes cluster within TAD and/or loops harboring differential CTCF and/ or H3K27ac peaks	7a	GLM Wald test	NA	*p-value<0.05, **p-value<0.01, ***p-value<0.001	
Significant changes in gene expression, CTCF and H3K27ac peaks in TADs and CTCF HiChIP loops	Supp 7a	DEseq2 Wald test	NA	FDR < 0.01	

Supplementary Table 2: statistical tests and cutoff used in this study

References

1. Whyte, W.A. et al. Master transcription factors and mediator establish super-enhancers at key cell identity genes. Cell 153, 307-19 (2013).

2. Bailey, T.L. et al. MEME SUITE: tools for motif discovery and searching. Nucleic Acids Res 37, W202-8 (2009).

3. Lazaris, C., Kelly, S., Ntziachristos, P., Aifantis, I. & Tsirigos, A. HiC-bench: comprehensive and reproducible Hi-C data analysis designed for parameter exploration and benchmarking. BMC Genomics 18, 22 (2017).

4. Nora, E.P. et al. Targeted Degradation of CTCF Decouples Local Insulation of Chromosome Domains from Genomic Compartmentalization. Cell 169, 930-944 e22 (2017).

5. Schwarzer, W. et al. Two independent modes of chromatin organization revealed by cohesin removal. Nature 551, 51-56 (2017).
