# Supplementary material for: NSD2 overexpression drives clustered chromatin and transcriptional changes in a subset of insulated domains
Source: Nat Commun. 2019 Oct 24;10:4843. doi: 10.1038/s41467-019-12811-4 (PMC6813313; doi:10.1038/s41467-019-12811-4)
Supplement: Supplementary file 3 — Description of Additional Supplementary Files [file 41467_2019_12811_MOESM3_ESM.pdf]

Description of Additional Supplementary Files

File Name: Supplementary Data 1
Description: Differentially expressed genes. Excel table showing for each gene, the output from DESeq2 analysis. Log2FC and -log10(FDR) are shown in the volcano plot in Fig. 1b.

File Name: Supplementary Data 2
Description: Differential H3K27ac peaks. Excel table showing for each H3K27ac peak, the output from DESeq2 analysis. Log2FC and -log10(FDR) are shown in the volcano plot in Fig. 1c.

File Name: Supplementary Data 3
Description: Differential CTCF peaks. Excel table showing for each CTCF peak, the output from DESeq2 analysis. Log2FC and -log10(FDR) are shown in the volcano plot in Fig. 2a.

File Name: Supplementary Data 4
Description: Logistic regression model matrix. Data frame showing for each gene, the output from the logistic regression model shown in Fig. 7a.
